# Quality of Media Reporting of Suicide in Nepal

**DOI:** 10.1155/2022/5708092

**Published:** 2022-07-07

**Authors:** Pawan Sharma, Ravi Raj Timasina, Swarndeep Singh, Shreeya Gyawali, Kedar Marahatta, S. M. Yasir Arafat

**Affiliations:** ^1^Patan Academy of Health Sciences, Lalitpur, Nepal; ^2^Gandaki Medical College Teaching Hospital, Pokhara, Nepal; ^3^All India Institute of Medical Sciences, New Delhi, India; ^4^North East London NHS Foundation Trust, UK; ^5^World Health Organization Country Office for Nepal, Kathmandu, Nepal; ^6^Enam Medical College and Hospital, Dhaka, Bangladesh

## Abstract

**Objectives:**

Suicide is a major public health concern. Sensible media reporting of suicide is one of the important prevention strategies. There has been no report assessing the quality of media reporting of suicide in Nepal. We aimed to assess the quality of newspaper reporting of suicide in Nepal against the World Health Organization (WHO) reporting guidelines.

**Methods:**

We undertook a content analysis study of articles from the online archives on reporting of suicide deaths in six English language (daily or weekly) newspapers published in Nepal over the two-year duration from a period between January 1, 2019, and December 31, 2020. Also, we compared them with the World Health Organization (WHO) guidelines.

**Results:**

A total of 165 English newspaper articles reporting on suicide were analyzed. 163 (98.8%) of news were published in the main section of the newspaper, and the mean length was 17.6 sentences. The name and age of the person who died of suicide were mentioned in about 69.1% (*n* = 114) and 53.3% (*n* = 88) articles, respectively. The most common method of suicide reported in the news articles was hanging (45.5%, *n* = 75), followed by poisoning (11.5%, *n* = 19). About 97.6% (*n* = 161) of news articles violated the recommendation provided in the WHO guidelines.

**Conclusions:**

The adherence to the WHO guidelines for media reporting of suicide in Nepal was found to be poor, with a large majority of news reports having at least one potentially harmful media characteristic. Only a small minority of news reports included potentially helpful information to prevent suicide.

## 1. Introduction

Media plays an important role in various aspects of suicide prevention. It can be an avenue of public education to promote help seeking, to reduce stigma related to mental health problems or suicide. Contrarily, it can glamorize suicide and promote suicide as a solution to problems in life [1]. Suicide is a major public health problem globally; and hence, reporting of suicide is a public health issue [2, 3]. With the increase in media broadcasting in recent years, the intensity and frequency of media reporting of suicide have also increased markedly [4]. The World Health Organization (WHO) and several other national and international organizations have released guidelines for promoting sensible media reporting on suicide and related topics (e.g., suicidal attempt) [5]. The available literature provides empirical evidence suggesting that responsible media reporting on suicidal behavior is one of the effective public health interventions in suicide prevention [6, 7]. Media blackouts on reporting of suicide have coincided with decreases in suicide rates [8]. A 1987 campaign to decrease media coverage of subway suicides in Austria has been shown to reduce subway suicide rates by about 80 percent [9].

Suicide is a key public health concern in Nepal. As per National Mental Health Survey of Nepal done in 2019-2020, the prevalence of suicidal thoughts among the adult population was 6.5% and prevalence of lifetime suicidal attempts was 1.1% [10]. The misconception that suicide is illegal and a punishable offence in Nepal is widely prevalent which has led to underreporting of cases [11]. News about suicide is commonly encountered in the national as well as local media. However, no guidelines have been formulated or implemented on media reporting of suicide in Nepal. In a country like Nepal where mental health issues are rarely discussed publicly due to stigma, media can play an important role in promoting appropriate help seeking behaviors, especially among people with suicidal thoughts [12].

Media portrayal of suicide in a sensational or dramatized manner has been routinely seen in the national news media. However, scientific studies to critically analyze the content and quality of news reports on suicide in Nepal have not been adequately conducted. Hence, the aim of current study is to perform content analysis of news reports published about suicide in national print media of Nepal and to assess adherence to the WHO guidelines on responsible media reporting of suicide.

## 2. Methods

We undertook a content analysis study of news articles retrieved from online archives which reported on suicide deaths. News articles from six English language (daily or weekly) newspapers published in Nepal between January 1, 2019 and December 31, 2020 were searched manually. The newspapers included were The Kathmandu Post, The Himalayan Times, Republica, The Rising Nepal, The Nepali Times, and The Annapurna Express. Two authors (SG and RT) independently reviewed and extracted relevant information using a predesigned format in Microsoft Excel.

All news articles in English that specifically reported suicide and suicide attempts of Nepalese people currently living in Nepal were included in the study. News articles covering suicide bombing, suicide terror attacks, and reports with controversy (reports where reason of death was not clearly established and being investigated by government agencies, i.e., suicide vs. homicide) were excluded.

First, descriptive information was extracted from each article, which included details like name of newspaper and length of article (i.e., number of sentences), whether or not the suicide event was connected to an instance of homicide–suicide or a suicide pact, among others. Second, the quality of articles was evaluated using a checklist prepared on the basis of the WHO responsible media reporting of suicide guidelines (see Supplementary file 1), similar to that used in previous studies [5, 13, 14]. The items were coded as “1” if the guideline was violated and “0” if the guideline was adhered to in the report. Any discrepancy or disagreement between the two researchers was resolved by consensus. Cross-checking was done to ensure the quality of data by the first author.

Descriptive statistics were used to analyze the data extracted from news articles using SPSS version 17.0 (IBM Corp, Armonk, NY).

No human subject was enrolled for this study, and publicly available news reports were extracted and analyzed; hence, no institutional ethical clearance was sought. However, the declaration of Helsinki was complied with in this research.

## 3. Results

A total of 165 English newspaper articles reporting on suicide were analyzed. A large majority were published in the main section of newspaper (*n* = 163, 98.8%). About 17% (*n* = 28) were published as front-page news reports. The news reports were kept brief, with a mean length of 17.6 sentences (median length = 14.0 sentences). This suggests that majority of news reports are brief in length and are likely to be inadequate to describe the complex issues surrounding death by suicide. A high proportion of articles reported upon suicide deaths linked to instances of homicide-suicide (*n* = 17, 10.3%) and suicide pacts (*n* = 8, 4.8%). Out of 165, 26 (15.8%) articles reported upon suicide death of a popular public figure (e.g., actor and politician). This suggests that responsible reporting of celebrity suicide could be an important strategy in reducing the risk of copycat suicides following suicide death by popular public figures. The names and age of person died of suicide were mentioned in about 69.1% (*n* = 114) and 53.3% (*n* = 88) articles, respectively. Someone was being either charged for abetment of suicide or being planned for the same in 10% (*n* = 17) articles. The most common method of suicide reported in the news articles was hanging (45.5%, *n* = 75), followed by poisoning (11.5%, *n* = 19).

The quality of news articles reporting suicide deaths was assessed following the WHO media reporting guidelines on suicide. The descriptive analysis of news articles for potentially harmful and helpful media report characteristics is described in Tables 1 and 2, respectively. About 97.6% (*n* = 161) of news articles violated the recommendation provided in the guideline, by including at least one potentially harmful information. The most common violations of the WHO media reporting guidelines were reporting upon the method of suicide used by the deceased and use of inappropriate terminology like “committed and commits” in suicide description, followed by providing a monocausal explanation for suicide. The most commonly included helpful information in news reports as per the WHO media guidelines was recognition of link between mental illness and suicide and reporting of suicide statistics, followed by inclusion of expert opinion about suicide by a mental health professional.

## 4. Discussion

This study is aimed at exploring the quality of suicide news reports published in English newspapers of Nepal against the WHO guidelines considering the importance of media strategies promoting responsible media reporting of suicide as one of the interventions to prevent suicide at the population level [15]. Almost all the reports were published in the main section of newspaper highlighting the newsworthiness of suicide in Nepal. The disclosure of personal details of the person dying by suicide seen in our study could be problematic, as suicide is still considered a stigmatizing condition in Nepalese society. The consequence of revealing such details could be increased stigmatization and/or discrimination against the family members of the deceased person [16, 17]. The media attention received by relatively rare events like suicide pacts and suicide-homicide as is much more than other common suicide incidents. This is likely due to such events suicide being considered as more sensational stories by news media professionals.

The available literature shows that media reporting of suicide is a double-edged sword. Sensational and/or inappropriate reporting of suicide could lead to increased copycat suicide and subsequent increase in suicide rates (known as the Werther effect) [18]. On the contrary, sensible media reporting of suicide with inclusion of educative and helpful information about suicide has been shown to reduce subsequent suicide rates, also known as Papageno effect [18]. Educative and helpful information could be in the form of sharing links of educative websites or resources and providing contact details for seeking help like suicide helplines [18].

In view of this, the present study finding that majority of news reports violated at least one recommendation of the WHO's responsible media reporting guidelines on suicide is particularly concerning. The mention of method, specific details, and place of suicide can influence vulnerable people such as children or adolescents going through a stressful period and other people with suicidal ideation and increase the risk of copycat or imitation suicide [1]. The monocausal explanations for suicide were provided highlighting cause effect relationship of negative life events in contrast to available scientific literature. Due to potentially harmful reporting false public opinion can be created and suicide may be portrayed as a potential solution to difficult life situations or problems (e.g., interpersonal conflicts and financial loss). Suicide has a complex, multifactorial causation, and the person with suicidal thoughts often shares the same with a general health care physician or close family/friend in the week preceding the actual attempt [19]. The high rates of inclusion of potentially harmful information in the news reports on suicide observed in this study are comparable with that reported from other available studies assessing the quality of media reporting on suicide from the South-East Asian region [20] and neighboring countries like India [21–24] or Bangladesh [14].

The language used for describing suicide was also found to be inappropriate and not in keeping with the WHO guidelines in majority of news reports. For example, about 62% of news reports used the c-words “committed,” “commits,” or “committing” while describing suicide. Additionally, about 4% of reports used the phrase “successful suicide,” suggesting death following suicide attempt as a favorable outcome. The WHO guidelines have recommended against the use of c-word, as the phrase “committed suicide” could inadvertently strengthen the public notions of linking suicide as a criminal act and/or a moral turpitude [25]. This could lead to increased stigmatization and moral condemnation of suicide (or suicide attempt) victims and their family members. Thus, use of nonstigmatizing language and appropriate terms (e.g., instead of “committed suicide,” use “died by suicide”; instead of “successful suicide,” use “ended/took his/her life,” etc.) while describing suicide has been advocated by several professional scientific organizations and researchers [26]. This is in contrast to the findings from a recent study from India, in which only a minority (18.5%) of media reports used the c-word. One possible explanation for this could be the positive effect of Press Council of India's guidelines on media reporting of suicide, which was released in September 2019 [23]. The low rates of inclusion of potentially helpful information in the news reports on suicide observed in this study are comparable with that reported from other available studies assessing the quality of media reporting on suicide from the South-East Asian region [20] and neighboring countries like India [21–23] or Bangladesh [13].

The overall low quality of media reporting on suicide observed in this study could be due to several possible reasons such as lack of awareness about the WHO guidelines on responsible media reporting of suicide, the pressure upon journalists to write sensational stories to increase readership, and involvement of crime reporters instead of health correspondents to cover suicide deaths, among others [27, 28].

In Nepalese context, there seems to be a wide variation in the way media reporting is carried out. We recommend a uniform national suicide reporting guideline for the entire media contextualizing the local sentiments and cultural aspects. There needs to be regular workshops involving mental health experts and media professionals to sensitize media personnel about the need and importance of adhering to sensible media reporting of suicide. There are several methods of encouraging the good media reporting. One of it could be instituting yearly awards or prizes to recognize journalists involved in creating awareness about suicide and suicide prevention through their work (e.g., Papageno Media Award) [29]. There is also a need to conduct more studies to assess the effectiveness of different strategies aimed at improving the quality of media reporting on suicide and also monitor the effect of media reporting and other suicide prevention strategies on actual population suicide rates and trends in a reliable and accurate manner to inform future policy and actions.

The current study is one of very few studies to systematically assess the quality of print news media reports on suicide from Nepal. The findings of the present study should be interpreted while keeping in mind certain important limitations. The major limitation is that the study included only print media reports published in English newspapers and did not include reports published in vernacular language. Only selected English newspapers were included. Thus, caution should be exercised while generalizing the present study findings to all media reporting in Nepal. Further, suicide news coverage on other media platforms such as social media, television, or radio were not assessed in this study. This is an important area for future research, as studies from other countries have reported that suicide news on television or social media (e.g., Twitter) were associated with increased suicide rates [30].

## 5. Conclusion

The adherence to the WHO guidelines for media reporting of suicide was found to be poor, with a large majority of news reports having at least one potentially harmful media characteristic. Moreover, only a small minority of new reports included potentially helpful information to prevent suicide. This study provides valuable baseline information about existing media practices and helps in identifying areas for improvement of media reporting on suicide in Nepal. There is an urgent need to develop national guidelines on media reporting of suicide and to conduct regular workshops with media professionals to educate them about these guidelines. Lastly, adequate training and support should be provided to media professionals for improving media reporting of suicide.

## Figures and Tables

**Table 1:** Potentially harmful media reporting characteristics according to World Health Organization suicide reporting guidelines (*N* = 165 articles).

Media report characteristics	Frequency (%)
Reporting of suicide method	114 (69.1%)
Use of “committed,” “commits,” or “committing” in suicide description	102 (61.8%)
Monocausal explanation for suicide	77 (46.7%)
Negative life events mentioned	67 (40.6%)
Word “suicide” in headline	67 (40.6%)
Detailed account of suicide method (i.e., at least two specific details about how the method was implemented)	45 (27.3%)
Sensational headline	42 (25.5%)
Highly prominent placement (front-page story)	28 (17.0%)
Life event(s) in headline	27 (16.4%)
Public site named as location of a suicide death	22 (13.3%)
Interview with bereaved persons	22 (13.3%)
Mention of suicide method in headline	19 (11.5%)
Details from suicide note reported	18 (10.9%)
Photo of the suicide deceased	17 (10.3%)
Step-by-step account of suicide method	12 (7.3%)
Suggests suicide to be out of the blue event (i.e., without any warning signs)	10 (6.1%)
Use of “successful suicide” term for completed suicide	7 (4.2%)
Use of sensational descriptors (e.g., “suicide epidemic,” “suicide capital”)	6 (3.6%)
Photo of the place or scene where of suicide	3 (1.8%)

**Table 2:** Potentially helpful media reporting characteristics according to World Health Organization suicide reporting guidelines (*N* = 165 articles).

Media report characteristics	Frequency (%)
Recognizes link of suicide with mental illness	48 (29.1%)
Suicide statistics reported	41 (24.8%)
Expert opinion from a mental health professional	32 (19.4%)
Mentions a suicide prevention program/support service	24 (14.5%)
Dispels myths regarding suicide (e.g., there are no preceding warning signs and/or that there is nothing one can do to prevent suicide)	21 (12.7%)
Scientific research findings reported	19 (11.5%)
Recognizes link of suicide with drug or alcohol abuse/dependence	14 (8.5%)
Provides contact details for a suicide support service (e.g., suicide helpline)	8 (4.8%)

## Data Availability

Data will be made available by the corresponding author on request to the email: pawan60@gmail.com.
